# *Klebsiella pneumoniae* Siderophores Induce Inflammation, Bacterial Dissemination, and HIF-1α Stabilization during Pneumonia

**DOI:** 10.1128/mBio.01397-16

**Published:** 2016-09-13

**Authors:** Victoria I. Holden, Paul Breen, Sébastien Houle, Charles M. Dozois, Michael A. Bachman

**Affiliations:** a Department of Microbiology and Immunology, University of Michigan, Ann Arbor, Michigan, USA; b Department of Pathology, University of Michigan, Ann Arbor, Michigan, USA; c Institut National de la Recherche Scientifique, Institut Armand-Frappier, Laval, Canada

## Abstract

*Klebsiella pneumoniae* is a Gram-negative pathogen responsible for a wide range of infections, including pneumonia and bacteremia, and is rapidly acquiring antibiotic resistance. *K. pneumoniae* requires secretion of siderophores, low-molecular-weight, high-affinity iron chelators, for bacterial replication and full virulence. The specific combination of siderophores secreted by *K. pneumoniae* during infection can impact tissue localization, systemic dissemination, and host survival. However, the effect of these potent iron chelators on the host during infection is unknown. *In vitro*, siderophores deplete epithelial cell iron, induce cytokine secretion, and activate the master transcription factor hypoxia inducible factor-1α (HIF-1α) protein that controls vascular permeability and inflammatory gene expression. Therefore, we hypothesized that siderophore secretion by *K. pneumoniae* directly contributes to inflammation and bacterial dissemination during pneumonia. To examine the effects of siderophore secretion independently of bacterial growth, we performed infections with *tonB* mutants that persist *in vivo* but are deficient in siderophore import. Using a murine model of pneumonia, we found that siderophore secretion by *K. pneumoniae* induces the secretion of interleukin-6 (IL-6), CXCL1, and CXCL2, as well as bacterial dissemination to the spleen, compared to siderophore-negative mutants at an equivalent bacterial number. Furthermore, we determined that siderophore-secreting *K. pneumoniae* stabilized HIF-1α *in vivo* and that bacterial dissemination to the spleen required alveolar epithelial HIF-1α. Our results indicate that siderophores act directly on the host to induce inflammatory cytokines and bacterial dissemination and that HIF-1α is a susceptibility factor for bacterial invasion during pneumonia.

## IMPORTANCE

*Klebsiella pneumoniae* causes a wide range of bacterial diseases, including pneumonia, urinary tract infections, and sepsis. To cause infection, *K. pneumoniae* steals iron from its host by secreting siderophores, small iron-chelating molecules. Classically, siderophores are thought to worsen infections by promoting bacterial growth. In this study, we determined that siderophore-secreting *K. pneumoniae* causes lung inflammation and bacterial dissemination to the bloodstream independently of bacterial growth. Furthermore, we determined that siderophore-secreting *K. pneumoniae* activates a host protein, hypoxia inducible factor (HIF)-1α, and requires it for siderophore-dependent bacterial dissemination. Although HIF-1α can protect against some infections, it appears to worsen infection with *K. pneumoniae*. Together, these results indicate that bacterial siderophores directly alter the host response to pneumonia in addition to providing iron for bacterial growth. Therapies that disrupt production of siderophores could provide a two-pronged attack against *K. pneumoniae* infection by preventing bacterial growth and preventing bacterial dissemination to the blood.

## INTRODUCTION

*Klebsiella pneumoniae* is a Gram-negative bacterium within the *Enterobacteriaceae* family and is the causative agent of a wide range of infections, including pneumonia, urinary tract infections, wound infections, and bacteremia. As the third-most-common cause of hospital-acquired infections, *K. pneumoniae* represents a major health care threat (1). Further compounding this concern, *K. pneumoniae* is rapidly acquiring resistance to all known antibiotics, thus becoming increasingly difficult to treat. In particular, carbapenem-resistant strains of *K. pneumoniae* are resistant to all or nearly all antibiotics and exhibit strikingly high mortality rates of 41% to 50% for bloodstream infections (2, 3).

In order to establish infection, *K. pneumoniae* secretes molecules called siderophores that are critical for bacterial growth and replication (4, 5). Siderophores are small, high-affinity iron-chelating molecules secreted by a wide variety of microorganisms that are critical for virulence in many Gram-negative bacteria (6). Enterobactin (Ent), with the highest known affinity for iron of any molecule, is the prototypic catecholate siderophore and effectively outcompetes host iron-binding proteins for iron (7). To counter the effects of Ent, neutrophils and epithelial cells secrete lipocalin 2 (Lcn2; also known as NGAL, Scn, and 24p3), which binds Ent with subnanomolar affinity (8). *In vivo*, the presence of Lcn2, by preventing bacterial uptake of Ent, has been shown to be bacteriostatic (9, 10). Bacteria have evolved to secrete Lcn2-evasive siderophores, such as salmochelin (Sal), a glycosylated Ent that requires the *iroA* locus for production and transport; the phenolate siderophore yersiniabactin (Ybt); and the citrate-hydroxamate siderophore aerobactin (6, 11, 12).

Because iron is critical for the function of many cellular processes, including DNA replication and oxygen metabolism, and as a cofactor for many cellular reactions, iron chelation by siderophores could have significant effects on host cells (13, 14). However, the effects of siderophore-dependent manipulation of host iron homeostasis during bacterial infection are largely unknown. Iron chelation by siderophores in the presence of Lcn2 induces *in vitro* proinflammatory cytokine secretion of interleukin-8 (IL-8), IL-6, and CCL20 from lung epithelial cells (15, 16). Siderophores also induce the stabilization of the master transcription factor hypoxia inducible factor-1α (HIF-1α) *in vitro* (15). HIF-1α regulates the expression of many genes, including those involved in glycolysis, inflammation, and angiogenesis, and is itself regulated by the availability of oxygen or iron within a cell (17–19). In normoxia, HIF-1α protein is targeted for degradation by prolyl hydroxylases, a reaction that requires iron (20). However, under conditions of low oxygen or low iron levels, HIF-1α protein is stabilized and translocates to the nucleus to activate gene expression (20–22). In addition to roles in adaptation to hypoxia and tumor development, HIF-1α activation has recently been associated with innate immunity against infections. Infection with *Pseudomonas aeruginosa* in a *Caenorhabditis elegans* model system identified siderophore-dependent activation of a hypoxic host response that was partially protective (23). In a murine urinary tract infection (UTI) model, HIF-1α was protective against *Escherichia coli* infection through host innate immunity modulation (24). These results indicate that siderophores have a broad and inflammatory effect on host epithelial cells in addition to their role as iron acquisition molecules for bacteria. However, a critical barrier to investigating this phenomenon *in vivo* is that siderophores allow bacterial proliferation, and an increase in bacterial CFU may indirectly increase inflammation, tissue damage, and bacterial dissemination.

Ferric siderophores are actively imported by bacteria through siderophore-specific outer membrane receptors that are dependent on the TonB-ExuB-ExuD energy transducing system, and *tonB* mutants secrete siderophores but cannot utilize them for growth (25). *K. pneumoniae tonB* mutants retain their antiphagocytic capsule and serum resistance but are unable to grow under iron-limited conditions (26). Upon inoculation, *K. pneumoniae tonB* mutants stimulate the host immune response but do not cause a productive infection.

In this study, we tested the hypothesis that *K. pneumoniae* siderophores disrupt iron homeostasis in host cells, leading to altered host responses to pneumonia. To study the effects of siderophore secretion independently of the indirect effects of siderophore-dependent bacterial growth, we employed *tonB* mutants that are capable of secreting siderophores but are not capable of utilizing them. By dissociating siderophore secretion from bacterial growth, we could directly compare levels of proinflammatory cytokine secretion, bacterial dissemination, and HIF-1α stabilization in response to infection with isogenic siderophore synthesis mutants at equivalent bacterial numbers.

## RESULTS

### Siderophores induce bacterial dissemination and lung inflammation.

**FIG 1:**
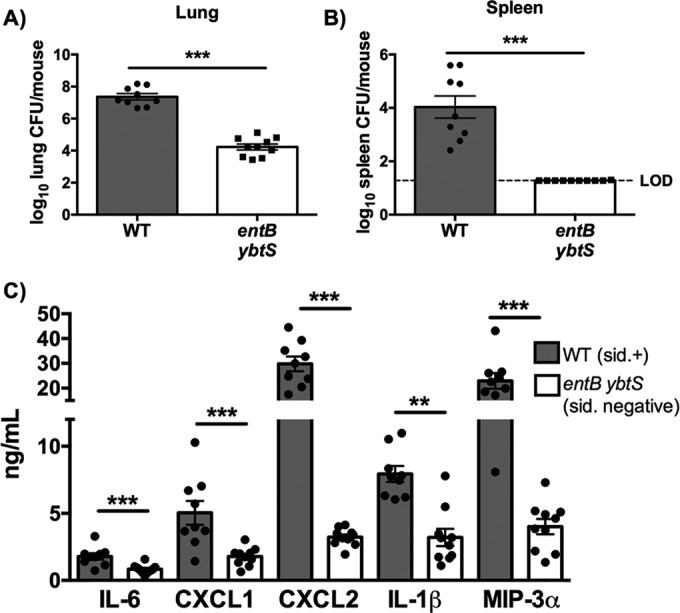
*K. pneumoniae* siderophores enhance bacterial growth and are required for cytokine secretion and bacterial dissemination. C57BL/6 mice (*n* = 9 to 10 per group) with 1 × 10^4^ CFU wild-type *K. pneumoniae* or *entB ybtS K. pneumoniae*, a mutant deficient in siderophore production. (A and B) At day 2, mice were euthanized, and organs were harvested for bacterial load in the (A) lung and (B) spleen. LOD, limit of detection. (C) Lung homogenates were assayed by ELISA for IL-6, CXCL1, CXCL2, IL-1β, and MIP-3α secretion. Statistics were calculated using *t*-test (A and B) or one-way ANOVA with Fisher’s posttest (**, *P* < 0.01; ***, *P* < 0.001 [as indicated]). sid., siderophore.

To measure the overall contribution of siderophores to inflammation and bacterial dissemination, mice were infected with a wild-type (WT) strain of *K. pneumoniae* that secretes Ent, Sal, and Ybt or an isogenic siderophore-negative *entB ybtS* mutant (4, 27). Infection with WT *K. pneumoniae* resulted in increased lung bacterial load compared to infection with the *entB ybtS* mutant (Fig. 1A). Furthermore, siderophores were required for *K. pneumoniae* dissemination to the spleen (Fig. 1B). Additionally, siderophores were required for induction of proinflammatory cytokines: the WT strain, but not the *entB ybtS* mutant, induced expression of interleukin-6 (IL-6), CXCL1, CXCL2, IL-1β, and macrophage inflammatory protein-3 alpha (MIP-3α) (Fig. 1C).

**FIG 2:**
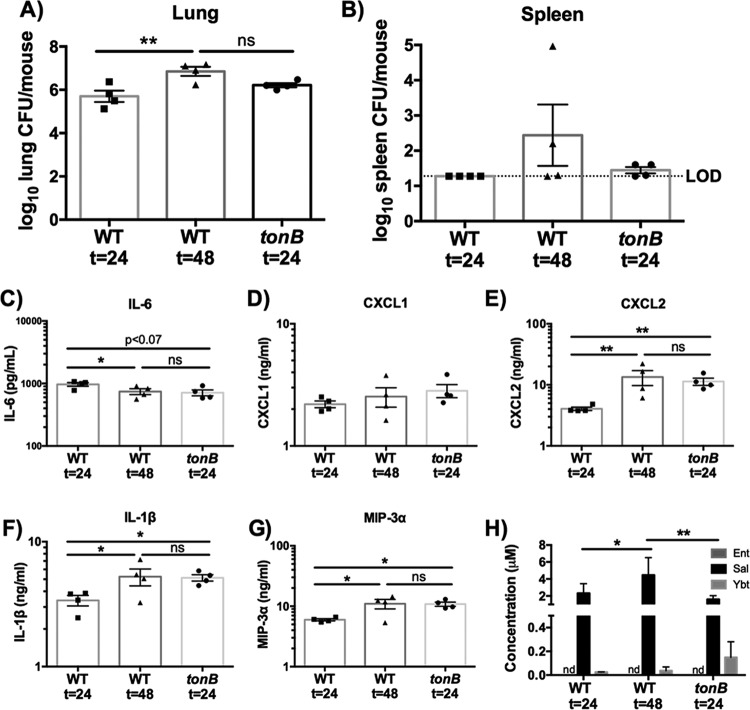
The results of 24-h *tonB K. pneumoniae* infection are comparable to those of 48-h wild-type infection. C57BL/6 mice (*n* = 4 per group) were infected with 1 × 10^4^ CFU wild-type *K. pneumoniae* or 1 × 10^8^ CFU *tonB K. pneumoniae*, a mutant that secretes but cannot take up siderophores. (A and B) Following 24 or 48 h, mice were euthanized, and organs were harvested for bacterial load in the (A) lung and (B) spleen. (C to H) Lung homogenates were assayed for (C) IL-6, (D) CXCL1, (E) CXCL2, (F) IL-1β, and (G) MIP-3α secretion by ELISA and (H) siderophore quantification by liquid chromatography-tandem mass spectrometry (LC-MS/MS). Statistics were calculated using one-way (A to G) or two-way (H) ANOVA with Fisher’s posttest (*, *P* < 0.05; **, *P* < 0.01; ns, *P* > 0.05). Elapsed time values represent hours.

The increase in bacterial dissemination and the inflammatory response during WT infection could be due to direct effects of iron chelation by siderophores on the host or to an indirect effect of siderophores increasing bacterial CFU in the lungs. To distinguish between these possibilities, we utilized a *tonB* mutant. As expected, the *tonB* mutant secreted siderophores but was not able to utilize endogenous or exogenous siderophores for bacterial growth (see Fig. S1 in the supplemental material). Because the WT and *tonB K. pneumoniae* strains produce an antiphagocytic capsule, we hypothesized that the *tonB* mutant would persist in the lung and secrete siderophores but would not replicate (26, 28). To compare infections performed with the WT and *tonB K. pneumoniae* strains, C57BL/6 mice were infected with 1 × 10^8^ CFU *tonB K. pneumoniae* or 1 × 10^4^ CFU WT *K. pneumoniae* for 24 or 48 h. After 24 h, *tonB K. pneumoniae* persisted in the lung and spleen, with a bacterial load comparable to that seen after 48 h of infection with WT *K. pneumoniae* (Fig. 2A and B). At 24 h, *tonB K. pneumoniae* infection also caused levels of induction of IL-6, CXCL1, CXCL2, IL-1β, and MIP-3α secretion that were comparable to those seen after 48 h of infection with WT *K. pneumoniae* (Fig. 2C to G). Additionally, we determined the concentrations of siderophores during infection with the *tonB* and WT *K. pneumoniae* strains by performing mass spectrometry (Fig. 2H; see also Fig. S2). Micromolar concentrations of Sal were detected in whole-lung homogenates from all infections. Ybt was detected in lower quantities in all lung samples, but Ent was not detected. Although bacterial growth dynamics differ, these results indicate that *tonB K. pneumoniae* infections can be used to examine the impact of siderophores on the host at concentrations and bacterial densities that mimic WT *K. pneumoniae* infection.

**FIG 3:**
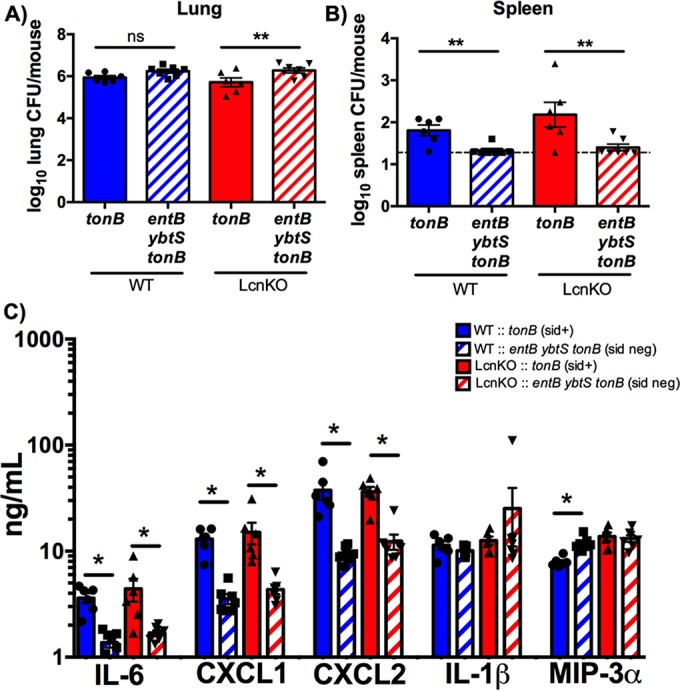
Siderophore secretion by *K. pneumoniae* results in bacterial dissemination and IL-6, CXCL1, and CXCL2 secretion in a Lcn2-independent manner. C57BL/6 mice (*n* = 6 to 7 per group) were infected with 1 × 10^8^ CFU *tonB* or *entB ybtS tonB K. pneumoniae*. (A and B) Following 24 h, mice were euthanized, and organs were harvested for bacterial load in the (A) lung and (B) spleen. (C) Lung homogenates were assayed for IL-1β, IL-6, CXCL1, CXCL2, and MIP-3α secretion by ELISA. Statistics were calculated using one-way ANOVA with Fisher’s posttest (*, *P* < 0.05; **, *P* < 0.01; ns, *P* > 0.05 [as indicated]).

To determine the impact of siderophore secretion on dissemination and cytokine responses, C57BL/6 mice were infected with isogenic *tonB* (siderophore-secreting) or *entB ybtS tonB* (siderophore-negative) *K. pneumoniae*. Despite equivalent bacterial loads, infection with *tonB K. pneumoniae* resulted in increased bacterial dissemination and IL-6, CXCL1, and CXCL2 secretion compared to infection with the *entB ybtS tonB* mutant (Fig. 3). Infection with the *tonB* strain did not enhance secretion of IL-1β or MIP-3α compared to the *entB ybtS tonB* mutant (Fig. 3C), suggesting that the differences between the level of induction by the WT strain and the level of induction by the *entB ybtS* strain were attributable to differences in bacterial density (Fig. 1C). *In vitro*, Lcn2 enhances induction of proinflammatory cytokines by siderophores (15). To examine the contribution of Lcn2, Lcn2-deficient (LcnKO) mice were infected with *tonB* or *entB ybtS tonB K. pneumoniae*. The same pattern of cytokine induction and dissemination was observed in WT and LcnKO mice, indicating that Lcn2 was not required for siderophore-dependent dissemination and inflammation *in vivo* (Fig. 3).

### Multiple siderophores are required for bacterial dissemination and cytokine secretion.

**FIG 4:**
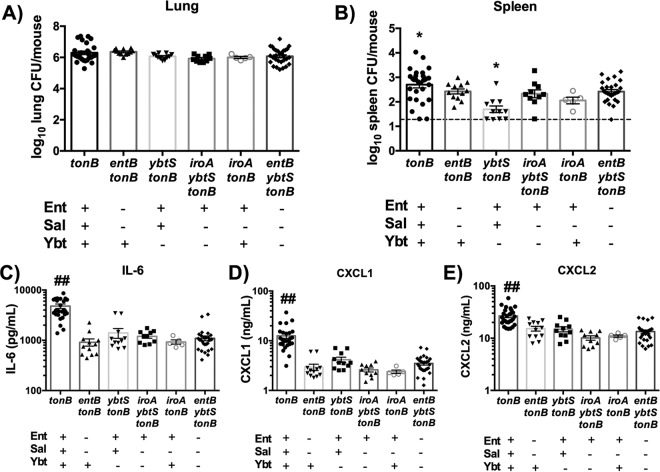
Multiple siderophores are required for bacterial dissemination and IL-6, CXCL1, and CXCL2 secretion. C57BL/6 mice (*n* = 5 to 18 per group) were infected with 1 × 10^8^ CFU isogenic *tonB K. pneumoniae* as indicated. (A and B) Following 24 h, mice were euthanized, and organs were harvested for bacterial load in the (A) lung and (B) spleen. (C to E) Lung homogenates were assayed for (C) IL-6, (D) CXCL1, and (E) CXCL2 secretion using ELISA. Statistics were calculated using one-way ANOVA with Fisher’s posttest (*, *P* < 0.05 [versus *entB ybtS tonB*]; ##, *P* < 0.001 [versus all other conditions]).

*In vitro*, Ent and Ybt induced proinflammatory cytokine secretion, but Sal did not (15). Because WT *K. pneumoniae* secretes Ent, Sal, and Ybt, it is unclear which siderophores are required to induce bacterial dissemination and inflammation during pneumonia. To test which siderophores were necessary for maximal dissemination and cytokine secretion, we used isogenic siderophore-secreting *tonB* mutants (Table 1) that secrete one or two siderophores to infect C57BL/6 mice. The *iroA* locus, required to produce Sal, was disrupted by mutation of the *iroB* glycosylase gene (9). Importantly, the infections performed with all mutants resulted in equivalent lung bacterial loads (Fig. 4A). Consistently, infection with *tonB K. pneumoniae* induced significantly more dissemination to the spleen than infection with the *entB ybtS tonB* mutant (Fig. 4B). No other siderophore mutant induced significant dissemination compared to the *entB ybtS tonB* (siderophore-negative) mutant. These data indicate that all siderophores are required in combination for maximal bacterial dissemination to the spleen.

**Table 1:** *K. pneumoniae* mutants used in this work

Strain	Description	Siderophore produced			Reference or source
		Ent	Ybt	Sal	
Wild type	KPPR1; RifR derivative of ATCC 43816	+	+	+	27
*entB ybtS*	VK089; KPPR1 *entB ybtS*	−	−	−	27
*tonB*	KP273; KPPR1 *tonB*::*kan*	+	+	+	This work
*entB ybtS tonB*	KP281; VK089 *tonB*::*kan*	−	−	−	This work
*entB tonB*	KP285; VK087 *tonB*::*kan*	−	+	−	This work
*ybtS tonB*	KP277; VK088 *tonB*::*kan*	+	−	+	This work
*iroA ybtS tonB*	KP2202; KP20 *tonB*::*hyg*	+	−	−	This work
*iroA tonB*	KP2227; KP25 *tonB*::*hyg*	+	+	−	This work

To determine if each individual siderophore is sufficient to induce cytokine secretion, enzyme-linked immunosorbent assays (ELISAs) were performed on lung homogenates taken at 24 h postinfection. Only infection with the *tonB* mutant was sufficient to induce more secretion of IL-6, CXCL1, or CXCL2 than infection with the *entB ybtS tonB* (siderophore-negative) mutant, indicating that all three siderophores are required for secretion of these cytokines (Fig. 4C to E). Consistent with Fig. 3, siderophore secretion did not specifically induce IL-1β or MIP-3α (see Fig. S3 in the supplemental material). Binding by Lcn2 could mask the effects of Ent on inflammation and dissemination. To test this hypothesis, we compared the levels of cytokine secretion and dissemination in C57BL/6 and LcnKO mice infected with *iroA ybtS tonB* (Ent-positive [Ent^+^]) *K. pneumoniae* (see Fig. S4). LcnKO mice did not display differences in spleen bacterial load or lung inflammation compared to C57BL/6 WT mice, indicating that the presence of Ent is not sufficient to induce dissemination or inflammation, even in the absence of Lcn2. Together, these results indicate that Sal, Ent, and Ybt are required to induce maximal secretion of IL-6, CXCL1, and CXCL2.

### Siderophores secreted by *K. pneumoniae* stabilize HIF-1α.

To test the hypothesis that *K. pneumoniae* siderophores stabilize HIF-1α during pneumonia, we utilized a transgenic mouse model that expresses a fusion protein of luciferase with the oxygen-dependent domain (ODD) (ODD-Luc) of HIF-1α that is subject to prolyl hydroxylation and becomes stabilized under low oxygen or low iron conditions (29–31). Infection of ODD-Luc mice with WT *K. pneumoniae* induced increased bioluminescence in the lung compared to the results obtained with a phosphate-buffered saline (PBS) vehicle control (see Fig. S5A in the supplemental material). To determine whether siderophores secreted by *K. pneumoniae* induce HIF-1α stabilization *in vivo* during infection, ODD-Luc mice were infected with *tonB* (siderophore-positive) or *entB ybtS tonB* (siderophore-negative) *K. pneumoniae*. Infection with *tonB* induced greater bioluminescence in the lung than infection with *entB ybtS tonB*, though infection with *entB ybtS tonB* did induce some bioluminescence compared to infection with the PBS vehicle control (see Fig. S5B). These results indicate that siderophores secreted *in vivo* can stabilize the HIF-1α transcription factor.

**FIG 5:**
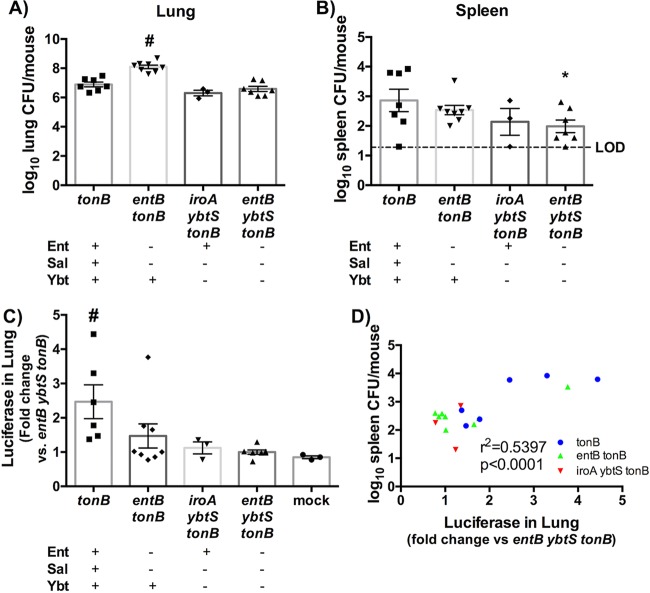
Siderophore secretion by *K. pneumoniae* induces HIF-1α stabilization, which correlates to bacterial dissemination to the spleen. ODD-luciferase mice (*n* = 3 to 8 per group) were infected with 1 × 10^8^ CFU isogenic *tonB K. pneumoniae* as indicated. (A to C) Following 24 h, mice were euthanized, and organs were harvested for (A) lung bacterial burden, (B) spleen bacterial burden, and (C) luciferase quantification. (D) Correlation curves were plotted comparing spleen CFU counts as a function of the level of luciferase in the lung. Statistics were calculated using one-way ANOVA with Fisher’s posttest (*, *P* < 0.05 [versus *tonB*]; #, *P* < 0.05 [versus all other conditions]) or the Pearson *r* correlation curve.

We then sought to determine whether individual siderophores were capable of stabilizing HIF-1α. To do so, ODD-Luc mice were infected with isogenic siderophore *tonB* mutants, and bacterial loads in the lung and spleen were quantified (Fig. 5A and B). The *tonB* mutants had equivalent lung CFU levels but significantly higher spleen CFU levels than the *entB ybtS tonB* mutants, consistent with previous data. Additionally, luciferase expression in the lung homogenate was quantified as fold change compared to the *entB ybtS tonB* (siderophore-negative) mutant. Infection with the *tonB* mutant induced significantly more luciferase expression than infection with the other isogenic strains (Fig. 5C). Infection with the *iroA ybtS tonB* (Ent^+^) strain resulted in CFU counts equivalent to *tonB* and *entB ybtS tonB* infection but did not result in significant dissemination to the spleen or induction of luciferase. ODD-Luc mice infected with the *entB tonB* (Ybt^+^) mutant displayed a higher bacterial load in the lung upon infection, confounding comparisons to the other strains. This mutant induced increased luciferase expression for a few mice and had correspondingly high CFU counts in the spleen. We therefore hypothesized that siderophore-dependent HIF-1α stabilization in the lung correlates with bacterial load in the spleen and that the outliers in the *entB tonB* mutant are the exceptions that prove the rule. To examine this hypothesis, spleen bacterial load was graphed as a function of luciferase levels in the lung, and the correlation was determined. This plot revealed a positive correlation between spleen bacterial load and luciferase expression during infection with *K. pneumoniae* across all siderophore mutant genotypes (Fig. 5D). Taken together, these data indicate that HIF-1α is consistently stabilized only by *tonB K. pneumoniae* that produces all three siderophores and that stabilization of HIF-1α by *K. pneumoniae* secreting siderophores correlates with bacterial dissemination to the spleen.

### Lung epithelial HIF-1α is required for bacterial dissemination to the spleen.

**FIG 6:**
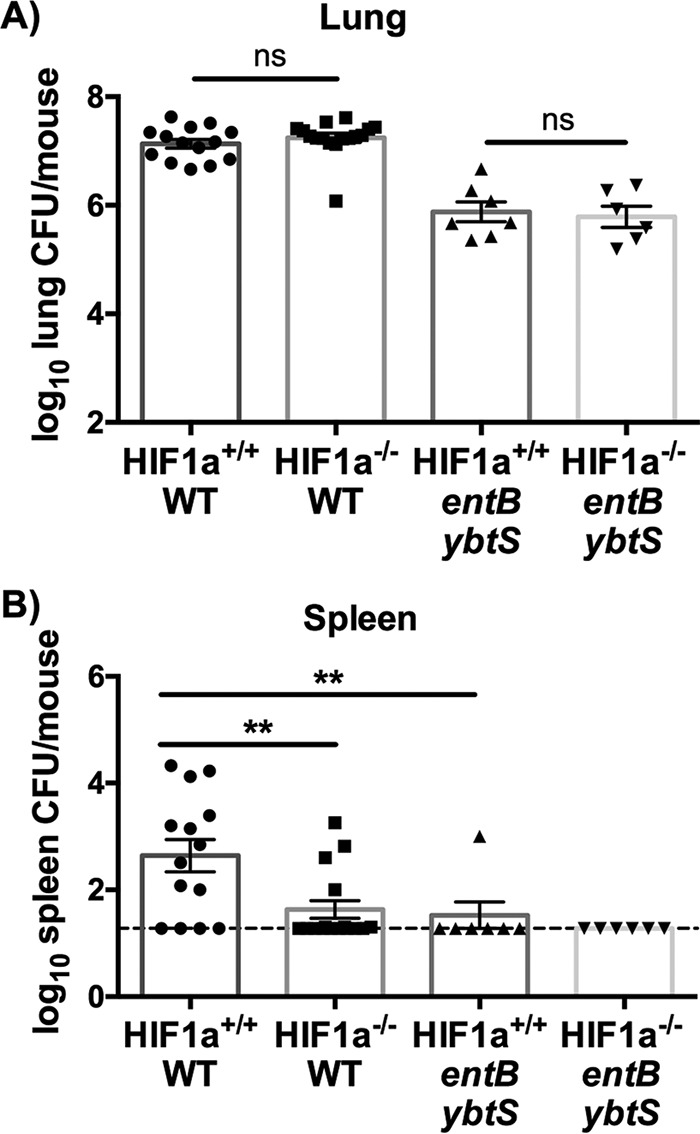
Lung epithelial HIF-1α is necessary for siderophore-dependent bacterial dissemination to the spleen. *Hif1a*^+/+^ or *Hif1a*^−/−^ mice (*n* = 6 to 14 per group) were infected with 1 × 10^4^ CFU wild-type or *entB ybtS K. pneumoniae*. Following 24 h, mice were euthanized, and organs were harvested for bacterial load in the (A) lung and (B) spleen. Statistics were calculated using one-way ANOVA with Fisher’s posttest (**, *P* < 0.01; ns, *P* > 0.05 [as indicated]).

Because HIF-1α stabilization correlates with bacterial dissemination to the spleen, and because HIF-1α regulates vascular permeability and inflammation, we hypothesized that HIF-1α is required for siderophore induction of bacterial dissemination and the host proinflammatory response. To test this hypothesis, we utilized transgenic mice that have an inducible lung epithelial cell-specific *Hif1a* deletion (55), using either mice induced with doxycycline postnatally (*Hif1a*^−/−^) or uninduced, wild-type littermates (*Hif1a*^+/+^). To test the effect of epithelial HIF-1α on a productive infection with replicative *K. pneumoniae*, the WT strain and *entB ybtS* mutant were used instead of their *tonB* counterparts. Infection with WT *K. pneumoniae* did not cause HIF-1α-dependent differences in lung bacterial load (Fig. 6A). However, *Hif1a*^−/−^ mice displayed significantly less bacterial dissemination to the spleen after 24 h, indicating that HIF-1α promotes bacterial dissemination to the spleen (Fig. 6B). The siderophore-negative *entB ybtS* mutant had lower lung and spleen CFU counts than the WT, which was not affected by the absence of lung epithelial HIF-1α (Fig. 6). Infection with WT *K. pneumoniae* induced significantly more IL-6 and CXCL2 than infection with the *entB ybtS* mutant, consistent with the siderophore-dependent effects observed as described above, and more IL-1β, which may be attributable to higher bacterial density (see Fig. S6 in the supplemental material). However, there were no HIF-1α-dependent differences in lung cytokine secretion, indicating that lung epithelial HIF-1α is not required to induce IL-1β, IL-6, CXCL1, CXCL2, or MIP-3α secretion during infection (see Fig. S6). These data suggest a role for epithelial HIF-1α stabilization by siderophores in the induction of bacterial dissemination during *K. pneumoniae* infection.

## DISCUSSION

*K. pneumoniae* is a Gram-negative bacterium that is rapidly acquiring resistance to all known antibiotics, including carbapenems. Developing novel therapies to combat antibiotic-resistant infections requires a more complete understanding of disease pathogenesis. To determine the effect of siderophores on the host response to infection, we utilized *tonB* mutants that allowed us to uncouple siderophore secretion from bacterial growth. We show that *K. pneumoniae* siderophores are a major trigger of the inflammation and bacterial dissemination induced during lung infection with *K. pneumoniae*, independently of their ability to deliver iron to bacteria. Additionally, we show that the induction of bacterial dissemination by siderophores requires master transcription factor HIF-1α in lung epithelial cells. These findings represent a novel function for bacterial siderophores in cytokine secretion and bacterial dissemination and a novel function for host master transcription factor HIF-1α as a susceptibility factor for the development of sepsis.

Our data indicate that siderophores induce dissemination through chelation of host cellular iron, leading to inactivation of iron-dependent prolyl hydroxylases and HIF-1α stabilization in lung epithelial cells. We have previously shown that purified siderophores deplete cellular iron in respiratory epithelial cells and stabilize HIF-1α and that activation of HIF-1α-dependent gene expression is abrogated by iron (15). The siderophore desferrioxamine is a canonical activator of HIF-1α, indicating that iron chelation is sufficient for stabilization (24). Accordingly, we demonstrate that the prolyl-containing ODD of HIF-1α is stabilized *in vivo* by siderophore-producing *K. pneumoniae*. The WT strain secretes Sal and Ybt *in vivo*, and both are required for maximal HIF-1α induction. Siderophore-dependent dissemination is blocked in a lung epithelial HIF-1α knockout mouse, indicating that this cell type mediates bacterial spread from the lung to the spleen. The mechanism is unknown and, since HIF-1α is a global transcriptional regulator, may be complex and multifactorial. For example, HIF-1α regulates cellular metabolism and survival and can induce vascular permeability and angiogenesis (32, 33) or disruption of epithelial barriers (34).

HIF-1α stabilization by siderophore-dependent iron chelation leading to bacteremia contrasts with the protective effect of HIF-1α stabilization by a pharmacological molecule, AKB-4924, during murine UTI (24). These contrasting results may be due to differences in both pathogen and model system. For instance, treatment with AKB-4924 prevented internalization of *E. coli* by uroepithelial cells. *K. pneumoniae* is not readily internalized by epithelial cells due to its capsule; therefore, preventing the uptake of bacteria through HIF-1α stabilization may not be an effective therapy against *K. pneumoniae* infection (35). Together, these data illustrate the complexity of HIF-pathogen interactions and highlight the importance of evaluating many various bacterial infections and model systems.

Mass spectrometry demonstrated that Sal and Ybt are the main siderophores produced during *K. pneumoniae* lung infection (Fig. 2H; see also Fig. S2 in the supplemental material). This analysis has been used to quantify Ent, Sal, and aerobactin in chicken air sacs during *E. coli* infection (36). To our knowledge, these data represent the first published concentrations of siderophores from murine lung homogenates. The measured concentrations were of the same magnitude as the concentrations used *in vitro* by our group, as well as others, and provide a context for prior findings obtained using purified siderophores (15, 16, 37). Although *tonB K. pneumoniae* secreted more siderophores *in vitro*, the concentrations observed *in vivo* were equal to or even slightly lower than those seen with the WT strain, indicating that the *tonB* mutants can assess the impact of siderophores on the host at physiologically relevant concentrations. Whereas micromolar concentrations of Sal and high nanomolar amounts of Ybt were detected, we were unable to detect Ent. We propose three possible explanations for this finding: (i) Ent is sequestered by Lcn2, and is therefore undetectable; (ii) bacteria convert all Ent to Sal *in vivo* to evade Lcn2; or (iii) a combination of our two hypotheses, whereby bacteria convert the majority of Ent to Sal and the remaining Ent is sequestered by Lcn2. These data suggest that the majority of inflammation and bacterial dissemination is due to the secretion of Sal but also suggest a role for Ybt. However, we could not test the contribution of Sal in isolation because it is not possible to create mutants that produce Sal without intact Ent synthesis genes, and we were unable to detect iron chelation by purified Sal *in vitro* (12, 15). In contrast, a mutant making Ent alone (the *iroA ybtS tonB* mutant) showed no detectable induction of cytokines or dissemination, with or without Lcn2. Together, these data implicate Sal and Ybt as significant inducers of inflammation and dissemination during pneumonia.

In addition to inducing dissemination, Ybt and Sal are required for maximal induction of IL-6, CXCL1, and CXCL2, which are all protective against lung infection with *K. pneumoniae* (38–40). These results are consistent with prior *in vitro* data illustrating that iron chelation by siderophores, as evidenced by depletion of the labile iron pool and induction of the iron starvation marker *NDRG1*, induces the secretion of proinflammatory cytokines IL-8 and IL-6 from A549 lung epithelial cells (15). Human IL-6 and murine IL-6 act as inflammatory cytokines involved in hepatocyte acute-phase responses and can upregulate hepcidin, an iron homeostasis protein (41, 42). Murine CXCL1 and CXCL2 are neutrophil chemoattractants and are functionally similar to human IL-8 (43). Whereas lung epithelial HIF-1α is instrumental in bacterial dissemination to the spleen, it was not required for the induction of cytokines. These results contrast with studies showing that HIF-1α regulates IL-6 and that epithelial HIF-1α regulates IL-6 and IL-1β secretion in a lung contusion model (44, 45). Myeloid cell HIF-1α has been shown to be instrumental in inflammation through myeloid cell development, phagocytosis, and antimicrobial production (46–48). In addition to HIF-1α regulation of inflammation, HIF-2α can regulate macrophage function in tumor models, eosinophil function in the lung, and IL-6 secretion from endothelial cells (49–51). Therefore, it is possible that another cell-specific HIF-1α or HIF-2α could be responsible for regulating cytokine secretion in response to *K. pneumoniae* infection. Because HIF knockouts are embryonically lethal, testing other HIF isoforms and cell types would require multiple lineage-specific knockouts.

Although Lcn2 was necessary for siderophore induction of cytokines *in vitro*, it was dispensable for the siderophore-dependent immune response to *K. pneumoniae* in vivo (15). This may indicate differential abilities of human and murine Lcn2 to modulate immune responses. Although they share 62% amino acid identity, murine Lcn2 lacks the ability to form covalent complexes, which may explain this discrepancy (52). Alternatively, redundant signaling pathways may be activated during pneumonia such that inflammatory signaling by Lcn2 is dispensable. It may be that two signals are required for the maximal induction of cytokine secretion in response to infection with siderophore-secreting bacteria: (i) perturbation of iron homeostasis by siderophores and (ii) signaling by an inflammatory protein(s), including Lcn2. *In vivo*, many proteins could satisfy the requirement of the second signal, such as inflammasome activation or Toll-like receptor signaling activation by capsule and lipopolysaccharide (53, 54).

On the basis of our data, we propose the following model: upon infection and iron starvation, *K. pneumoniae* produces and secretes siderophores. Siderophores serve to acquire host iron and deliver it to the bacteria, resulting in bacterial growth. In addition to supporting bacterial growth, chelation of host cellular iron by siderophores induces cellular stress. One stress response is the stabilization of HIF-1α, ultimately resulting in bacterial dissemination to the spleen. An opposing stress response is secretion within the lung of the proinflammatory cytokines IL-6, CXCL1, and CXCL2, which are necessary for protection from *K. pneumoniae*. These results indicate novel functions for bacterial siderophores during infection that are independent of their iron delivery capabilities and present siderophore molecules as a possible target for therapeutic intervention. Additionally, these results indicate a novel role for HIF-1α as a susceptibility factor for systemic spread during *K. pneumoniae* infection and illustrate the complex interplay between pathogen and host molecules during bacterial infection.

## MATERIALS AND METHODS

### Animal strains and ethics statement.

All work was approved by the University of Michigan Institutional Animal Care and Use Committee (IACUC). C57BL/6 lipocalin 2-deficient (LcnKO), ODD-luciferase (ODD-Luc) (29), and conditional alveolar epithelial HIF-1α-deficient [SP-C-rtTA^−/tg^/(tetO)_7_-CMV-Cre^tg/tg^/HIF-1^flox/flox^] mice were bred onsite. To induce epithelial cell knockout of HIF-1α, newborn mice were treated as previously described (56).

### Bacterial strains and media.

*K. pneumoniae* KPPR1 and isogenic mutants were cultured in Luria-Bertani broth (LB) at 37°C with shaking or 30°C on agar (Becton, Dickinson and Company, Sparks, MD) supplemented with kanamycin (25 µg/ml), rifampin (30 µg/ml), or hygromycin (100 µg/ml) as indicated (57). As noted, descriptions of bacteria were obtained under iron-limited conditions: bacteria were grown overnight in LB; subcultured 1:100 and incubated for 2 h with 10 µM 2,2′-dipyridyl (DIP) at 37°C; subcultured into M9 media with 10^8^ CFU; and incubated overnight.

### Murine pneumonia model.

C57BL/6, lipocalin2-deficient (LcnKO) ODD-luciferase (ODD-Luc) *Hif1a*^−/−^ or *Hif1a*^+/+^ mice (6 to 10 weeks old) were infected with 1 × 10^4^ WT or 1 × 10^8^ CFU of indicated isogenic *tonB K. pneumoniae* mutant grown under iron-limited conditions as previously described (5). To determine bacterial numbers in tissues, whole lungs and spleens were removed and homogenized into 1 ml Dulbecco’s phosphate-buffered saline (DPBS) containing EDTA-free protease inhibitor (Roche) and cultured to obtain bacterial counts.

### ELISA.

Cytokine protein concentrations in lung homogenates were determined by ELISAs (Duoset kits; R&D Systems) according to the protocols of the manufacturer.

### Siderophore quantification in lung homogenates.

Whole lungs were collected and homogenized in 1 ml DPBS with protease inhibitors as described above, passed through a 0.2-µM-pore-size syringe filter (EMD Millipore, Darmstadt, Germany) to remove bacteria, and frozen at −80°C until analysis. Siderophore concentrations were determined via mass spectrometry as previously described (12, 36); complete experimental details can be found in Text S1 in the supplemental material.

### Luciferase assay.

ODD-Luc mice were infected and euthanized as described above, and lungs and spleens were collected. Lungs were homogenized with DPBS and protease inhibitor (Roche), and an aliquot was reserved for luciferase quantification. Luciferase cell lysis buffer (New England Biolabs, Ipswich, MA) was added to form a homogenate, and the reaction mixture was incubated at room temperature for 15 min. Protein concentrations were quantified using the bicinchoninic acid (BCA) assay (Thermo, Fisher). Thirty micrograms of protein was added to an opaque Corning 96-well plate (Corning, NY), and luciferase buffer was added using a BioTek Synergy multimode plate reader (BioTek). Luciferase buffer was composed of 4.8 ml 0.11 mM Tris (pH 7.8), 50 µl 100 mM sodium luciferin, 60 µl 200 mM ATP, and 120 µl 0.5 M MgCl_2_.

### Mutant construction.

PCR primers specific for conserved regions of the *tonB* gene were constructed by comparing DNA sequences from various *K. pneumoniae* species (see Table S1 in the supplemental material) (9). An internal 0.3-kb *tonB* PCR fragment was amplified and then cloned into TA-based PCR cloning vector pCR2.1 (Invitrogen, Carlsbad, CA). The *tonB* fragment was then extracted using a gel extraction kit (Qiagen, Venlo, Limburg, The Netherlands), purified with a PCR cleanup kit (Qiagen), dephosphorylated, and ligated with a kanamycin-resistant derivative of λpir-dependent suicide vector pGP704 (58). This *tonB* suicide vector was transformed into *E. coli* strain BW20767 [ATCC 47084; RP4-2tet::Mu-1kan::Tn*7* integrant *uidA*(DMlu1)::pir^+^ *recA1 creB510 leu-63 hsdR17 endA1 zbf-5 thi*] and subsequently conjugated into the wild-type strain and *entB*, *ybtS*, and *entB ybtS* mutants of *K. pneumoniae* to generate *tonB*, *entS tonB*, *ybtS tonB*, and *entB ybtS tonB* mutants. Integration of the suicide vector into the *tonB* gene was confirmed by generation of a PCR product using one primer on the vector (pGP704 MCS Pst.Xba) and a *tonB*-specific primer flanking the insertion site. To generate *tonB* mutants in *iroA* and *iroA ybtS* mutant backgrounds, Lambda Red mutagenesis of *tonB* was performed as previously described (5). The *iroA* and *iroA ybtS* mutants contained a cointegration of pGP704 in the *iroB* glycosylase gene that disrupts function of the *iroA* (salmochelin synthesis) locus as previously described (9). Primers are listed in Table S1.

### Statistical analysis.

Bacterial counts and ELISA data were log-transformed and analyzed using one-way analysis of variance (ANOVA) models with one mean per group, and pairs of treatments were compared with Fisher’s posttest (GraphPad Software, Inc.). Luciferase assay data were analyzed using one-way ANOVA with Fisher’s posttest. Correlation data were calculated using the Pearson r correlation curve.

### Data availability.

All data have been summarized in graphs shown in the main manuscript and supplemental figures.

## SUPPLEMENTAL MATERIAL

Figure S1  Mutants in *tonB* secrete siderophores but cannot take up siderophores for growth. (A) Bacteria were inoculated in LB overnight in a 96-well plate and grown overnight. Readings of optical density at 600 nm (OD_600_) were taken every 15 min. (B) Bacteria were grown overnight in LB media and then subcultured into a 96-well plate with RPMI–10% heat-inactivated (HI) serum overnight to determine the strain’s ability to grow under iron-limiting conditions. Strains were supplemented with exogenous Ent to examine if the siderophore could rescue growth. (C) Bacterial strains were grown overnight in M9 minimal media. Supernatants were spun through a 0.2-μm filter to remove bacteria, and iron-chelating molecules were assayed via the chrome azurol S (CAS) assay. Statistics were calculated using one-way ANOVA with Fisher’s posttest (***, *P* < 0.001 [versus *entB ybtS tonB*]; ##, *P* < 0.01; ###, *P* < 0.001 [as indicated]). Download Figure S1, TIF file, 1.7 MB

Figure S2  Sample LC-MS/MS traces from a lung homogenate and control siderophore extracts. The top four graphs show the multiple reaction monitoring (MRM) channels for salmochelins (linear monoglucosyl enterobactin [MGE]), yersiniabactin, and the internal standard PQS-D4 (5,6,7,8-tetradeutero-2-heptyl-3-hydroxy-4-quinolone) from a mouse infected with wild-type *K. pneumoniae*. The bottom four graphs represent control siderophore extracts. Download Figure S2, TIF file, 3.1 MB

Figure S3  IL-1β and MIP-3α during lung infection with isogenic *tonB* mutants. C57BL/6 mice (*n* = 5 to 18 per group) were infected with 1 × 10^8^ CFU isogenic *tonB K. pneumoniae* as indicated. Lung homogenates were assayed for (A) IL-1β and (B) MIP-3α secretion using ELISA. Download Figure S3, TIF file, 0.3 MB

Figure S4  Lcn2 does not impact dissemination or inflammation by Ent-secreting *tonB K. pneumoniae*. C57BL/6 or LcnKO mice (*n* = 4 to 5 per group) were infected with 1 × 10^8^ CFU *iroA ybtS tonB K. pneumoniae*. (A and B) Following a 24-h infection, mice were euthanized, and organs were harvested for bacterial load in the (A) lung and (B) spleen. (C to G) Lung homogenates were assayed by ELISA for (C) IL-6, (D) CXCL1, (E) CXCL2, (F) IL-1β, and (G) MIP-3α secretion. There were no statistically significantly differences in the assay results, as calculated using unpaired, two-tailed *t* tests. Download Figure S4, TIF file, 0.2 MB

Figure S5  Wild-type *K. pneumoniae* and *tonB K. pneumoniae* induce HIF-1α stabilization in the lung as indicated by bioluminescence. ODD-luciferase mice were infected with (A) 1 × 10^4^ CFU wild-type *K. pneumoniae* or (B) 1 × 10^8^ CFU *tonB* or *entB ybtS tonB K. pneumoniae* for 24 h*.* Mice were treated with luciferin and euthanized, and lungs were removed to image bioluminescence (photons per second per centimeter squared per steradian). Bioluminescence intensity is indicated with blue (representing low induction) and red (indicating high induction). Each well contained lungs from a single mouse. Data shown are representative of results from 3 to 7 individual mice. Download Figure S5, TIF file, 1.1 MB

Figure S6  Lung epithelial HIF-1α is not necessary for siderophore-dependent secretion of cytokines. HIF-1a^+/+^ or HIF-1a^−/−^ mice (*n* = 6 to 14 per group) were infected with 1 × 10^4^ CFU wild-type or *entB ybtS K. pneumoniae*. Following 24 h, mice were euthanized, and organs were harvested. Lung homogenates were assayed for (A) IL-6, (B) CXCL1, (C) CXCL2, (D) IL-1β, and (E) MIP-3α secretion by ELISA. Statistics were calculated using one-way ANOVA with Fisher’s posttest (*, *P* < 0.05; **, *P* < 0.01; ***, *P* < 0.001; ns, *P* > 0.05 [as indicated]). Download Figure S6, TIF file, 0.2 MB

Table S1  Primers used for mutagenesis in this work. Table S1, TIF file, 1.8 MB

Text S1  Supplemental methods. Download Text S1, DOC file, 0.2 MB

## References

[1] Magill SS, Edwards JR, Bamberg W, Beldavs ZG, Dumyati G, Kainer MA, Lynfield R, Maloney M, McAllister-Hollod L, Nadle J, Ray SM, Thompson DL, Wilson LE, Fridkin SK, Emerging Infections Program Healthcare-Associated Infections and Antimicrobial Use Prevalence Survey Team 2014 Multistate point-prevalence survey of health care-associated infections. N Engl J Med 370:1198–1208. doi:10.1056/NEJMoa1306801. 24670166 PMC4648343

[2] Munoz-Price LS, Poirel L, Bonomo RA, Schwaber MJ, Daikos GL, Cormican M, Cornaglia G, Garau J, Gniadkowski M, Hayden MK, Kumarasamy K, Livermore DM, Maya JJ, Nordmann P, Patel JB, Paterson DL, Pitout J, Villegas MV, Wang H, Woodford N, Quinn JP 2013 Clinical epidemiology of the global expansion of *Klebsiella pneumoniae* carbapenemases. Lancet Infect Dis 13:785–796. doi:10.1016/S1473-3099(13)70190-7. 23969216 PMC4673667

[3] Tumbarello M, Viale P, Viscoli C, Trecarichi EM, Tumietto F, Marchese A, Spanu T, Ambretti S, Ginocchio F, Cristini F, Losito AR, Tedeschi S, Cauda R, Bassetti M 2012 Predictors of mortality in bloodstream infections caused by *Klebsiella pneumoniae* carbapenemase-producing *K. pneumoniae*: importance of combination therapy. Clin Infect Dis 55:943–950. doi:10.1093/cid/cis588. 22752516

[4] Lawlor MS, O’Connor C, Miller VL 2007 Yersiniabactin is a virulence factor for *Klebsiella pneumoniae* during pulmonary infection. Infect Immun 75:1463–1472. doi:10.1128/IAI.00372-06. 17220312 PMC1828572

[5] Bachman MA, Breen P, Deornellas V, Mu Q, Zhao L, Wu W, Cavalcoli JD, Mobley HL 2015 Genome-wide identification of *Klebsiella pneumoniae* fitness genes during lung infection. mBio 6:e00775. doi:10.1128/mBio.00775-15. 26060277 PMC4462621

[6] Holden VI, Bachman MA 2015 Diverging roles of bacterial siderophores during infection. Metallomics 7:986–995. doi:10.1039/c4mt00333k. 25745886

[7] Crumbliss AL, Harrington JM 2009 Iron sequestration by small molecules: thermodynamic and kinetic studies of natural siderophores and synthetic model compounds. Adv Inorg Chem 61:179–250. doi:10.1016/S0898-8838(09)00204-9.

[8] Goetz DH, Holmes MA, Borregaard N, Bluhm ME, Raymond KN, Strong RK 2002 The neutrophil lipocalin NGAL is a bacteriostatic agent that interferes with siderophore-mediated iron acquisition. Mol Cell 10:1033–1043. doi:10.1016/S1097-2765(02)00708-6. 12453412

[9] Bachman MA, Miller VL, Weiser JN 2009 Mucosal lipocalin 2 has pro-inflammatory and iron-sequestering effects in response to bacterial enterobactin. PLoS Pathog 5:e1000622. doi:10.1371/journal.ppat.1000622. 19834550 PMC2757716

[10] Flo TH, Smith KD, Sato S, Rodriguez DJ, Holmes MA, Strong RK, Akira S, Aderem A 2004 Lipocalin 2 mediates an innate immune response to bacterial infection by sequestrating iron. Nature 432:917–921. doi:10.1038/nature03104. 15531878

[11] Fischbach MA, Lin H, Zhou L, Yu Y, Abergel RJ, Liu DR, Raymond KN, Wanner BL, Strong RK, Walsh CT, Aderem A, Smith KD 2006 The pathogen-associated *iroA* gene cluster mediates bacterial evasion of lipocalin 2. Proc Natl Acad Sci U S A 103:16502–16507. doi:10.1073/pnas.0604636103. 17060628 PMC1637611

[12] Bachman MA, Oyler JE, Burns SH, Caza M, Lépine F, Dozois CM, Weiser JN 2011 *Klebsiella pneumoniae* yersiniabactin promotes respiratory tract infection through evasion of lipocalin 2. Infect Immun 79:3309–3316. doi:10.1128/IAI.05114-11. 21576334 PMC3147564

[13] Pantopoulos K, Porwal SK, Tartakoff A, Devireddy L 2012 Mechanisms of mammalian iron homeostasis. Biochemistry 51:5705–5724. doi:10.1021/bi300752r. 22703180 PMC3572738

[14] Gkouvatsos K, Papanikolaou G, Pantopoulos K 2012 Regulation of iron transport and the role of transferrin. Biochim Biophys Acta 1820:188–202. doi:10.1016/j.bbagen.2011.10.013. 22085723

[15] Holden VI, Lenio S, Kuick R, Ramakrishnan SK, Shah YM, Bachman MA 2014 Bacterial siderophores that evade or overwhelm lipocalin 2 induce hypoxia inducible factor 1alpha and proinflammatory cytokine secretion in cultured respiratory epithelial cells. Infect Immun 82:3826–3836. doi:10.1128/IAI.01849-14. 24980968 PMC4187820

[16] Nelson AL, Ratner AJ, Barasch J, Weiser JN 2007 Interleukin-8 secretion in response to a ferric enterobactin is potentiated by siderocalin. Infect Immun 75:3160–3168. doi:10.1128/IAI.01719-06. 17420239 PMC1932857

[17] Palazon A, Goldrath AW, Nizet V, Johnson RS 2014 HIF transcription factors, inflammation, and immunity. Immunity 41:518–528. doi:10.1016/j.immuni.2014.09.008. 25367569 PMC4346319

[18] Lu H, Forbes RA, Verma A 2002 Hypoxia-inducible factor 1 activation by aerobic glycolysis implicates the Warburg effect in carcinogenesis. J Biol Chem 277:23111–23115. doi:10.1074/jbc.M202487200. 11943784

[19] Pugh CW, Ratcliffe PJ 2003 Regulation of angiogenesis by hypoxia: role of the HIF system. Nat Med 9:677–684. doi:10.1038/nm0603-677. 12778166

[20] Huang LE, Gu J, Schau M, Bunn HF 1998 Regulation of hypoxia-inducible factor 1alpha is mediated by an O2-dependent degradation domain via the ubiquitin-proteasome pathway. Proc Natl Acad Sci U S A 95:7987–7992. doi:10.1073/pnas.95.14.7987. 9653127 PMC20916

[21] Wang GL, Jiang BH, Rue EA, Semenza GL 1995 Hypoxia-inducible factor 1 is a basic-helix-loop-helix-PAS heterodimer regulated by cellular O2 tension. Proc Natl Acad Sci U S A 92:5510–5514. doi:10.1073/pnas.92.12.5510. 7539918 PMC41725

[22] Peyssonnaux C, Zinkernagel AS, Schuepbach RA, Rankin E, Vaulont S, Haase VH, Nizet V, Johnson RS 2007 Regulation of iron homeostasis by the hypoxia-inducible transcription factors (HIFs). J Clin Invest 117:1926–1932. doi:10.1172/JCI31370. 17557118 PMC1884690

[23] Kirienko NV, Kirienko DR, Larkins-Ford J, Wählby C, Ruvkun G, Ausubel FM 2013 *Pseudomonas aeruginosa* disrupts *Caenorhabditis elegans* iron homeostasis, causing a hypoxic response and death. Cell Host Microbe 13:406–416. doi:10.1016/j.chom.2013.03.003. 23601103 PMC3641844

[24] Lin AE, Beasley FC, Olson J, Keller N, Shalwitz RA, Hannan TJ, Hultgren SJ, Nizet V 2015 Role of hypoxia inducible factor-1alpha (HIF-1alpha) in innate defense against uropathogenic *Escherichia coli* infection. PLoS Pathog 11:e1004818. doi:10.1371/journal.ppat.1004818. 25927232 PMC4415805

[25] Noinaj N, Guillier M, Barnard TJ, Buchanan SK 2010 TonB-dependent transporters: regulation, structure, and function. Annu Rev Microbiol 64:43–60. doi:10.1146/annurev.micro.112408.134247. 20420522 PMC3108441

[26] Hsieh PF, Lin TL, Lee CZ, Tsai SF, Wang JT 2008 Serum-induced iron-acquisition systems and TonB contribute to virulence in *Klebsiella pneumoniae* causing primary pyogenic liver abscess. J Infect Dis 197:1717–1727. doi:10.1086/588383. 18433330

[27] Bachman MA, Lenio S, Schmidt L, Oyler JE, Weiser JN 2012 Interaction of lipocalin 2, transferrin, and siderophores determines the replicative niche of *Klebsiella pneumoniae* during pneumonia. mBio 3:e00224-11. doi:10.1128/mBio.00224-11. 23169997 PMC3509427

[28] Podschun R, Ullmann U 1992 *Klebsiella* capsular type K7 in relation to toxicity, susceptibility to phagocytosis and resistance to serum. J Med Microbiol 36:250–254. doi:10.1099/00222615-36-4-250. 1560447

[29] Safran M, Kim WY, O’Connell F, Flippin L, Günzler V, Horner JW, Depinho RA, Kaelin WG Jr. 2006 Mouse model for noninvasive imaging of HIF prolyl hydroxylase activity: assessment of an oral agent that stimulates erythropoietin production. Proc Natl Acad Sci U S A 103:105–110. doi:10.1073/pnas.0509459103. 16373502 PMC1324998

[30] Xue X, Ramakrishnan S, Anderson E, Taylor M, Zimmermann EM, Spence JR, Huang S, Greenson JK, Shah YM 2013 Endothelial PAS domain protein 1 activates the inflammatory response in the intestinal epithelium to promote colitis in mice. Gastroenterology 145:831–841. doi:10.1053/j.gastro.2013.07.010. 23860500 PMC3799890

[31] Zampell JC, Yan A, Avraham T, Daluvoy S, Weitman ES, Mehrara BJ 2012 HIF-1alpha coordinates lymphangiogenesis during wound healing and in response to inflammation. FASEB J 26:1027–1039. doi:10.1096/fj.11-195321. 22067482 PMC3470728

[32] Forsythe JA, Jiang BH, Iyer NV, Agani F, Leung SW, Koos RD, Semenza GL 1996 Activation of vascular endothelial growth factor gene transcription by hypoxia-inducible factor 1. Mol Cell Biol 16:4604–4613. doi:10.1128/MCB.16.9.4604. 8756616 PMC231459

[33] Weis SM, Cheresh DA 2005 Pathophysiological consequences of VEGF-induced vascular permeability. Nature 437:497–504. doi:10.1038/nature03987. 16177780

[34] Shah YM, Ito S, Morimura K, Chen C, Yim SH, Haase VH, Gonzalez FJ 2008 Hypoxia-inducible factor augments experimental colitis through an MIF-dependent inflammatory signaling cascade. Gastroenterology 134:2036–2048.e3. doi:10.1053/j.gastro.2008.03.009. 18439915 PMC2533811

[35] Sahly H, Podschun R, Oelschlaeger TA, Greiwe M, Parolis H, Hasty D, Kekow J, Ullmann U, Ofek I, Sela S 2000 Capsule impedes adhesion to and invasion of epithelial cells by *Klebsiella pneumoniae*. Infect Immun 68:6744–6749. doi:10.1128/IAI.68.12.6744-6749.2000. 11083790 PMC97775

[36] Caza M, Lépine F, Milot S, Dozois CM 2008 Specific roles of the *iroBCDEN* genes in virulence of an avian pathogenic *Escherichia coli* O78 strain and in production of salmochelins. Infect Immun 76:3539–3549. doi:10.1128/IAI.00455-08. 18541653 PMC2493193

[37] Chaturvedi KS, Hung CS, Crowley JR, Stapleton AE, Henderson JP 2012 The siderophore yersiniabactin binds copper to protect pathogens during infection. Nat Chem Biol 8:731–736. doi:10.1038/nchembio.1020. 22772152 PMC3600419

[38] Sutherland RE, Olsen JS, McKinstry A, Villalta SA, Wolters PJ 2008 Mast cell IL-6 improves survival from *Klebsiella pneumoniae* and sepsis by enhancing neutrophil killing. J Immunol 181:5598–5605. doi:10.4049/jimmunol.181.8.5598. 18832718 PMC2610024

[39] Cai S, Batra S, Lira SA, Kolls JK, Jeyaseelan S 2010 CXCL1 regulates pulmonary host defense to *Klebsiella* infection via CXCL2, CXCL5, NF-kappaB, and MAPKs. J Immunol 185:6214–6225. doi:10.4049/jimmunol.0903843. 20937845 PMC2974054

[40] Greenberger MJ, Strieter RM, Kunkel SL, Danforth JM, Laichalk LL, McGillicuddy DC, Standiford TJ 1996 Neutralization of macrophage inflammatory protein-2 attenuates neutrophil recruitment and bacterial clearance in murine Klebsiella pneumoniae. J Infect Dis 173:159–165. doi:10.1093/infdis/173.1.159. 8537653

[41] Wrighting DM, Andrews NC 2006 Interleukin-6 induces hepcidin expression through STAT3. Blood 108:3204–3209. doi:10.1182/blood-2006-06-027631. 16835372 PMC1895528

[42] Tanabe O, Akira S, Kamiya T, Wong GG, Hirano T, Kishimoto T 1988 Genomic structure of the murine IL-6 gene. High degree conservation of potential regulatory sequences between mouse and human. J Immunol 141:3875–3881. 3263439

[43] Zlotnik A, Yoshie O 2000 Chemokines: a new classification system and their role in immunity. Immunity 12:121–127. doi:10.1016/S1074-7613(00)80165-X. 10714678

[44] Yan SF, Tritto I, Pinsky D, Liao H, Huang J, Fuller G, Brett J, May L, Stern D 1995 Induction of interleukin 6 (IL-6) by hypoxia in vascular cells. Central role of the binding site for nuclear factor-IL-6. J Biol Chem 270:11463–11471. doi:10.1074/jbc.270.19.11463. 7744784

[45] Suresh MV, Ramakrishnan S, Thomas B, Machado-Aranda D, Bi Y, Talarico N, Anderson E, Shah YM, Raghavendran K 2014 Activation of hypoxia-inducible factor 1α in type 2 alveolar epithelial cell is a major driver of acute inflammation following lung contusion. Crit Care Med 42:e642–e653. doi:10.1097/CCM.0000000000000488. 25014067 PMC4245055

[46] Sickinger S, Maier H, König S, Vallant N, Kofler M, Schumpp P, Schwelberger H, Hermann M, Obrist P, Schneeberger S, Margreiter R, Troppmair J, Pratschke J, Aigner F 2013 Lipocalin-2 as mediator of chemokine expression and granulocyte infiltration during ischemia and reperfusion. Transpl Int 26:761–769. doi:10.1111/tri.12116. 23701109

[47] Cramer T, Yamanishi Y, Clausen BE, Förster I, Pawlinski R, Mackman N, Haase VH, Jaenisch R, Corr M, Nizet V, Firestein GS, Gerber HP, Ferrara N, Johnson RS 2003 HIF-1alpha is essential for myeloid cell-mediated inflammation. Cell 112:645–657. doi:10.1016/S0092-8674(03)00154-5. 12628185 PMC4480774

[48] Peyssonnaux C, Datta V, Cramer T, Doedens A, Theodorakis EA, Gallo RL, Hurtado-Ziola N, Nizet V, Johnson RS 2005 HIF-1alpha expression regulates the bactericidal capacity of phagocytes. J Clin Invest 115:1806–1815. doi:10.1172/JCI23865. 16007254 PMC1159132

[49] Imtiyaz HZ, Williams EP, Hickey MM, Patel SA, Durham AC, Yuan LJ, Hammond R, Gimotty PA, Keith B, Simon MC 2010 Hypoxia-inducible factor 2alpha regulates macrophage function in mouse models of acute and tumor inflammation. J Clin Invest 120:2699–2714. doi:10.1172/JCI39506. 20644254 PMC2912179

[50] Proper SP, Saini Y, Greenwood KK, Bramble LA, Downing NJ, Harkema JR, Lapres JJ 2014 Loss of hypoxia-inducible factor 2 alpha in the lung alveolar epithelium of mice leads to enhanced eosinophilic inflammation in cobalt-induced lung injury. Toxicol Sci 137:447–457. doi:10.1093/toxsci/kft253. 24218148 PMC3908723

[51] Endler A, Chen L, Li Q, Uchida K, Hashimoto T, Lu L, Xu GT, Shibasaki F 2013 Int6/eIF3e silenced HIF2alpha stabilization enhances migration and tube formation of HUVECs via IL-6 and IL-8 signaling. Cytokine 62:115–122. doi:10.1016/j.cyto.2013.01.021. 23478175

[52] Kjeldsen L, Cowland JB, Borregaard N 2000 Human neutrophil gelatinase-associated lipocalin and homologous proteins in rat and mouse. Biochim Biophys Acta 1482:272–283. doi:10.1016/S0167-4838(00)00152-7. 11058768

[53] Hua KF, Yang FL, Chiu HW, Chou JC, Dong WC, Lin CN, Lin CY, Wang JT, Li LH, Chiu HW, Chiu YC, Wu SH 2015 Capsular polysaccharide is involved in NLRP3 inflammasome activation by *Klebsiella pneumoniae* serotype K1. Infect Immun 83:3396–3409. doi:10.1128/IAI.00125-15. 26077758 PMC4534678

[54] Regueiro V, Moranta D, Campos MA, Margareto J, Garmendia J, Bengoechea JA 2009 *Klebsiella pneumoniae* increases the levels of Toll-like receptors 2 and 4 in human airway epithelial cells. Infect Immun 77:714–724. doi:10.1128/IAI.00852-08. 19015258 PMC2632040

[55] Saini Y, Harkema JR, LaPres JJ 2008 HIF1alpha is essential for normal intrauterine differentiation of alveolar epithelium and surfactant production in the newborn lung of mice. J Biol Chem 283:33650–33657. doi:10.1074/jbc.M805927200. 18801745 PMC2586263

[56] Saini Y, Kim KY, Lewandowski R, Bramble LA, Harkema JR, Lapres JJ 2010 Role of hypoxia-inducible factor 1alpha in modulating cobalt-induced lung inflammation. Am J Physiol Lung Cell Mol Physiol 298:L139–L147. doi:10.1152/ajplung.00252.2009. 19915160 PMC2822559

[57] Broberg CA, Wu W, Cavalcoli JD, Miller VL, Bachman MA 2014 Complete genome sequence of *Klebsiella pneumoniae* strain ATCC 43816 KPPR1, a rifampin-resistant mutant commonly used in animal, genetic, and molecular biology studies. Genome Announc 2:e00924-14. doi:10.1128/genomeA.00924-14. 25291761 PMC4175196

[58] Miller VL, Mekalanos JJ 1988 A novel suicide vector and its use in construction of insertion mutations: osmoregulation of outer membrane proteins and virulence determinants in *Vibrio cholerae* requires *toxR*. J Bacteriol 170:2575–2583. 2836362 10.1128/jb.170.6.2575-2583.1988 PMC211174

