# Supplementary figures and images for: Klebsiella pneumoniae Siderophores Induce Inflammation, Bacterial Dissemination, and HIF-1α Stabilization during Pneumonia
Source: mBio. 2016 Sep 13;7(5):e01397-16. doi: 10.1128/mBio.01397-16 (PMC5021805; doi:10.1128/mBio.01397-16)

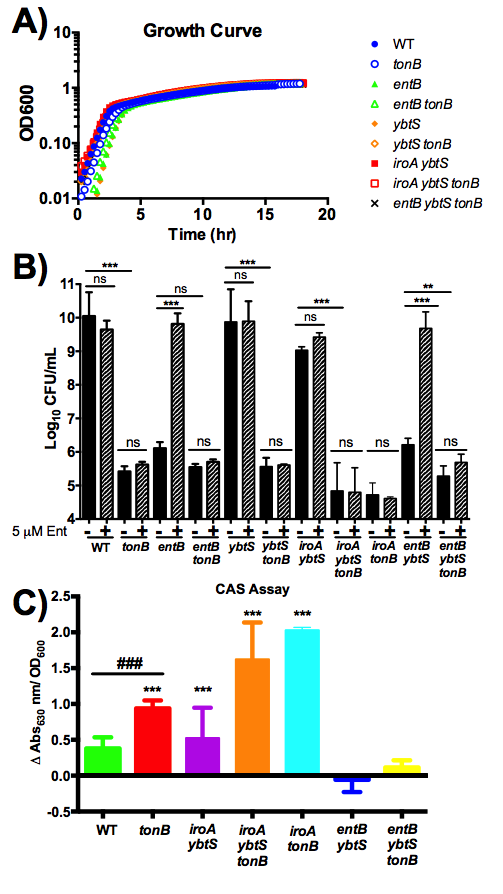

Supplement: Figure S1 — Mutants in tonB secrete siderophores but cannot take up siderophores for growth. (A) Bacteria were inoculated in LB overnight in a 96-well plate and grown overnight. Readings of optical density at 600 nm (OD600) were taken every 15 min. (B) Bacteria were grown overnight in LB media and then subcultured into a 96-well plate with RPMI–10% heat-inactivated (HI) serum overnight to determine the strain’s ability to grow under iron-limiting conditions. Strains were supplemented with exogenous Ent to examine if the siderophore could rescue growth. (C) Bacterial strains were grown overnight in M9 minimal media. Supernatants were spun through a 0.2-μm filter to remove bacteria, and iron-chelating molecules were assayed via the chrome azurol S (CAS) assay. Statistics were calculated using one-way ANOVA with Fisher’s posttest (***, P < 0.001 [versus entB ybtS tonB]; ##, P < 0.01; ###, P < 0.001 [as indicated]). Download [file mbo006162987sf1.tif]

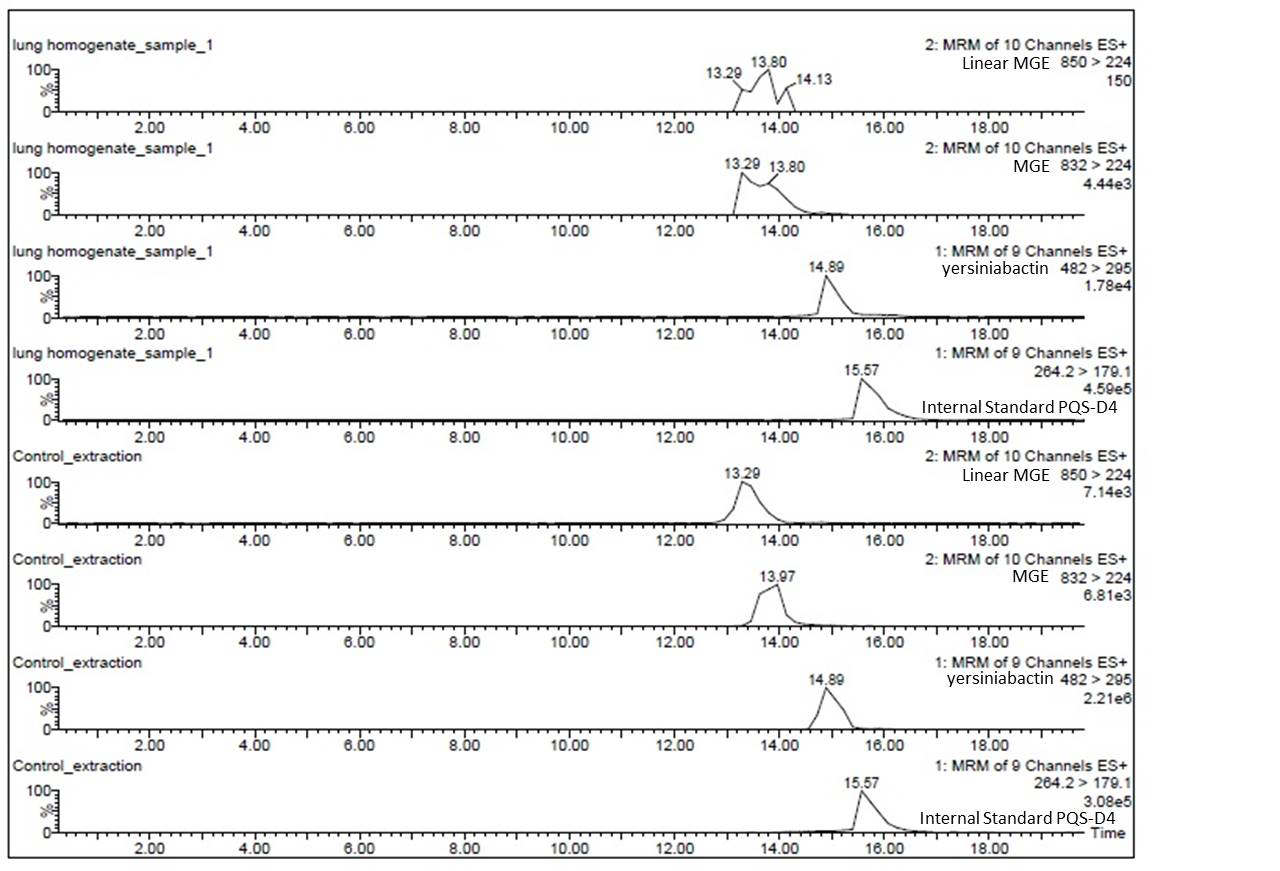

Supplement: Figure S2 — Sample LC-MS/MS traces from a lung homogenate and control siderophore extracts. The top four graphs show the multiple reaction monitoring (MRM) channels for salmochelins (linear monoglucosyl enterobactin [MGE]), yersiniabactin, and the internal standard PQS-D4 (5,6,7,8-tetradeutero-2-heptyl-3-hydroxy-4-quinolone) from a mouse infected with wild-type K. pneumoniae. The bottom four graphs represent control siderophore extracts. Download [file mbo006162987sf2.tif]

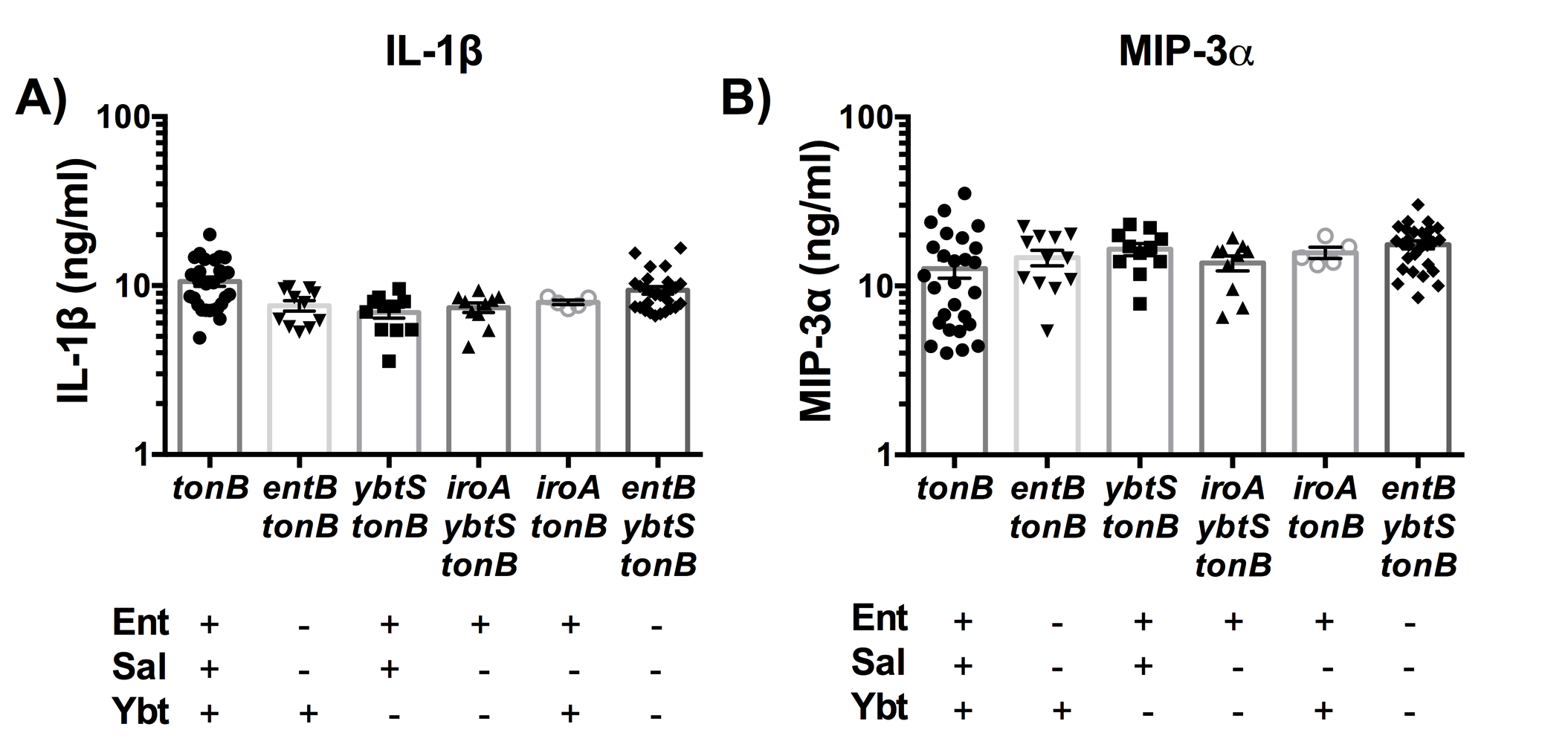

Supplement: Figure S3 — IL-1β and MIP-3α during lung infection with isogenic tonB mutants. C57BL/6 mice (n = 5 to 18 per group) were infected with 1 × 10^8^ CFU isogenic tonB K. pneumoniae as indicated. Lung homogenates were assayed for (A) IL-1β and (B) MIP-3α secretion using ELISA. Download [file mbo006162987sf3.tif]

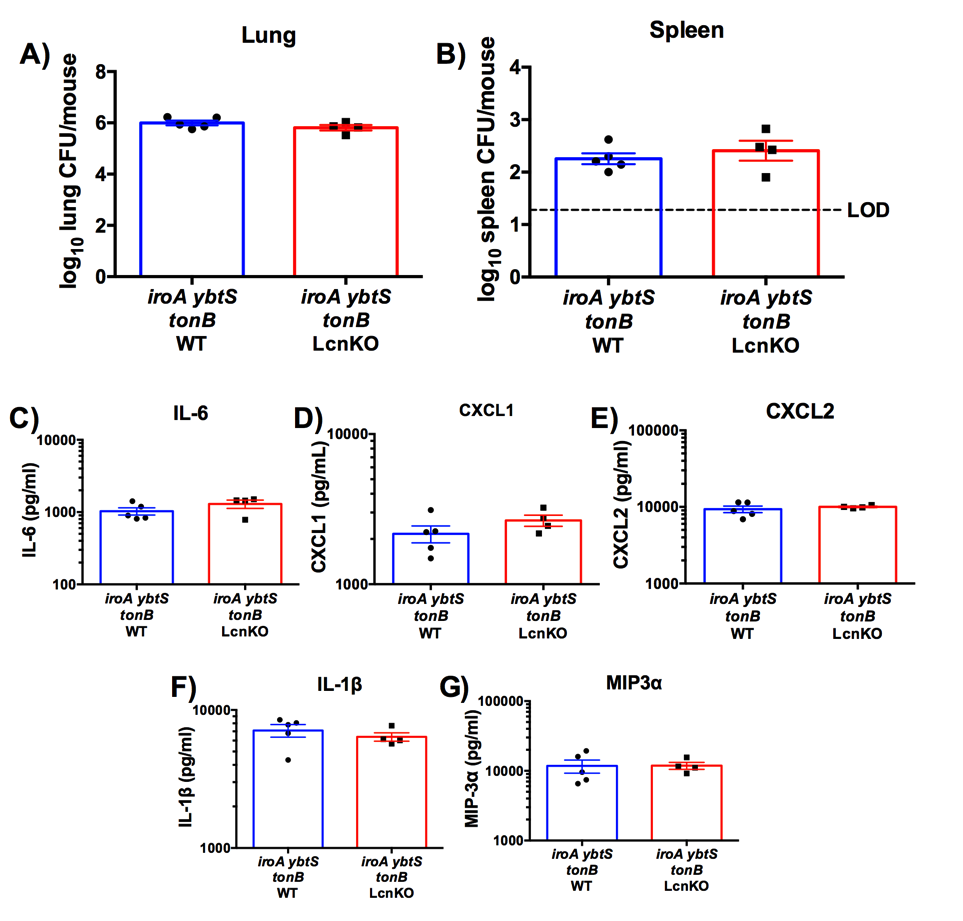

Supplement: Figure S4 — Lcn2 does not impact dissemination or inflammation by Ent-secreting tonB K. pneumoniae. C57BL/6 or LcnKO mice (n = 4 to 5 per group) were infected with 1 × 10^8^ CFU iroA ybtS tonB K. pneumoniae. (A and B) Following a 24-h infection, mice were euthanized, and organs were harvested for bacterial load in the (A) lung and (B) spleen. (C to G) Lung homogenates were assayed by ELISA for (C) IL-6, (D) CXCL1, (E) CXCL2, (F) IL-1β, and (G) MIP-3α secretion. There were no statistically significantly differences in the assay results, as calculated using unpaired, two-tailed t tests. Download [file mbo006162987sf4.tif]

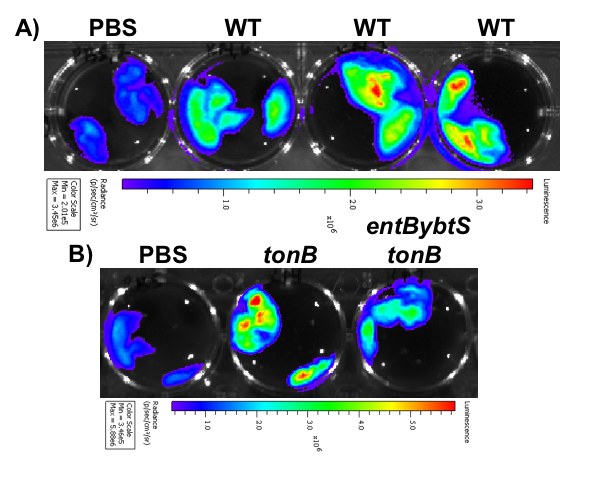

Supplement: Figure S5 — Wild-type K. pneumoniae and tonB K. pneumoniae induce HIF-1α stabilization in the lung as indicated by bioluminescence. ODD-luciferase mice were infected with (A) 1 × 10^4^ CFU wild-type K. pneumoniae or (B) 1 × 10^8^ CFU tonB or entB ybtS tonB K. pneumoniae for 24 h. Mice were treated with luciferin and euthanized, and lungs were removed to image bioluminescence (photons per second per centimeter squared per steradian). Bioluminescence intensity is indicated with blue (representing low induction) and red (indicating high induction). Each well contained lungs from a single mouse. Data shown are representative of results from 3 to 7 individual mice. Download [file mbo006162987sf5.tif]

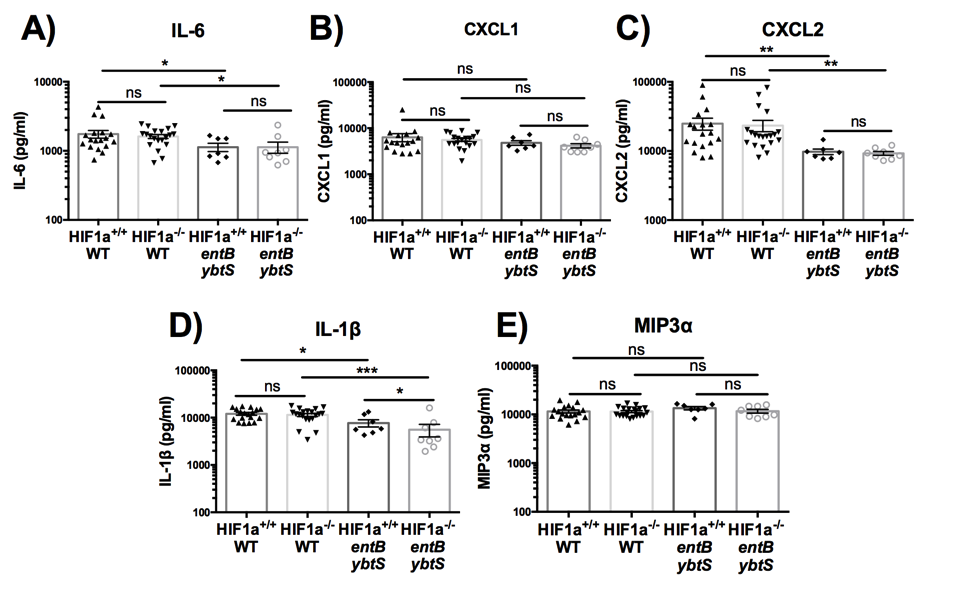

Supplement: Figure S6 — Lung epithelial HIF-1α is not necessary for siderophore-dependent secretion of cytokines. HIF-1a+/+ or HIF-1a−/− mice (n = 6 to 14 per group) were infected with 1 × 10^4^ CFU wild-type or entB ybtS K. pneumoniae. Following 24 h, mice were euthanized, and organs were harvested. Lung homogenates were assayed for (A) IL-6, (B) CXCL1, (C) CXCL2, (D) IL-1β, and (E) MIP-3α secretion by ELISA. Statistics were calculated using one-way ANOVA with Fisher’s posttest (*, P < 0.05; **, P < 0.01; ***, P < 0.001; ns, P > 0.05 [as indicated]). Download [file mbo006162987sf6.tif]

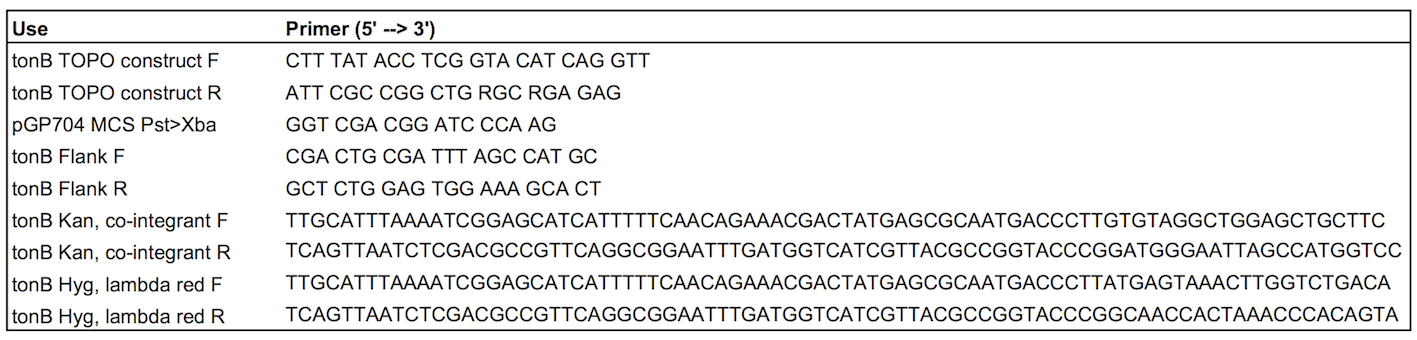

Supplement: Table S1 — Primers used for mutagenesis in this work. [file mbo006162987st1.tif]
