# Supplementary material for: Klebsiella pneumoniae Siderophores Induce Inflammation, Bacterial Dissemination, and HIF-1α Stabilization during Pneumonia
Source: mBio. 2016 Sep 13;7(5):e01397-16. doi: 10.1128/mBio.01397-16 (PMC5021805; doi:10.1128/mBio.01397-16)
Supplement: Text S1 — Supplemental methods. Download [file mbo006162987s1.doc]

**Supplemental Methods**

*Siderophore Quantification in lung homogenates.* Briefly, homogenates were centrifuged at 3,200×g for 15 min. The supernatants were retained and 500 μL aliquots were then prepared with 5,6,7,8-tetradeutero-2-heptyl-3-hydroxy-4-quinolone as an internal control. Samples were analyzed three times by LC-MS/MS.

Multiple reaction monitoring (MRM) analyses were performed using a Waters 2795 Alliance HT instrument coupled to a Micromass Quattro Premier XE spectrometer (Micromass MS Technologies). Samples were injected onto a Kinetex 2.6-μm C8 4.6- by 100-mm column at a flow rate of 400 μl/min, with a linear gradient of water-acetonitrile with 1% acetic acid. The high-performance liquid chromatography effluent was directed to the mass spectrometer through a Valco T splitter. The analyses were performed in positive electrospray ionization mode with a cone voltage of 30 V. Monitoring of daughter ions from specific pseudomolecular ions was performed by collision-induced dissociation with argon at different collision energies for each molecule ranging from 15-55 eV. The specific transition ions monitored from pseudomolecular ions to daughter ions of salmochelins SX, S1, linear diglucosyl-C-enterobactin (DGE) (S2), DGE (S4), S5, monoglucosyl-C-enterobactin (MGE), linear MGE, triglucosyl-C-enterobactin (TGE), and linear TGE were 404 > 299, 627 > 224, 1,012 > 224, 994 > 224, 789 > 386, 832 > 224, 850 > 224, 1,156 > 266 and 1,174 > 266 m/z, respectively. The transition ions monitored for enterobactin and its linear trimer [(DHBS)3], dimer [(DHBS)2], and monomer (DHBS) derivatives were 670 > 224, 688 > 224, 465 > 224, and 242 > 137 m/z, respectively. The transition ions monitored for the internal standard, aerobactin and yersiniabactin were 264 > 179, 565 > 205 and 482 > 295 m/z, respectively. Quantification of each compound was determined from the response factor of Ent for Sal and Ent corrected with the intensity of the signal of the internal standard. Quantification of Ybt was determined from the response factor of Ybt corrected with the intensity of the signal of the internal standard.

*Growth Curves.* *K. pneumoniae* strains were grown overnight in LB or M9 minimal media. On the following day cultures were incubated in LB with a starting density of 2.6x10^7^ CFU/ml and grown for 8 hours at 37^o^ C. OD600 readings were taken every 15 min using an Eon microplate spectrophotometer with Gen5 software (BioTek, Winooski, VT).

*Serum Growth Assay*. To assess the ability of *K. pneumoniae* mutants to acquire iron, serum growth assays were performed as previously described .

*CAS Assay.* Following overnight growth, cultures of *K. pneumoniae* were diluted 1:100 and incubated for 2 hours with 10 uM 2,2′-dipyridyl (DIP) at 37^o^ C, subcultured into M9 media with 10^8^ CFU, and incubated overnight. The following day, cultures were spun through 0.22 μm tube filters, and the supernatants were used for the CAS assay. CAS reagent was prepared as previously described . Briefly, 2 mM of chrome azurol S (CAS) agent was prepared. Next, iron (III) solution (1mM FeCl into 10 mM of HCl), 3 mM hexadecyltrimethrylammonium bromide (HDTMA) solution, and 1 mM anhydrous piperazine solution were prepared (Sigma). 7.5 mL of 10 M HCl was added to the piperazine solution, and 1.5 mL of the iron (III) solution was added to 7.5 mL of the 2 mM CAS solution. Finally, the acid-piperazine solution was poured into the iron-CAS-HDTMA solution, and the final volume was brought to 100 mL with ddH_2_O (pH 5.6).

*Bioluminescence.* To determine the effect of *K. pneumoniae* infection on HIF-1α stabilization, six- to ten-week old ODD-Luc mice were infected as above for 24 hours. Following infection, mice were treated with 100 μL luciferin intraperitonally and euthanized as above. Lungs were removed for bioluminescent imaging and imaged on an IVIS bioluminescent imaging system at the Center for Molecular Imaging at the University of Michigan.
